# High Resolution Genetic and Physical Mapping of a Major Powdery Mildew Resistance Locus in Barley

**DOI:** 10.3389/fpls.2019.00146

**Published:** 2019-02-14

**Authors:** Parastoo Hoseinzadeh, Ruonan Zhou, Martin Mascher, Axel Himmelbach, Rients E. Niks, Patrick Schweizer, Nils Stein

**Affiliations:** ^1^Department of Genebank, Leibniz Institute of Plant Genetics and Crop Plant Research, Gatersleben, Germany; ^2^Department of Plant Science, Plant Breeding, Wageningen University & Research, Wageningen, Netherlands; ^3^Department of Crop Sciences, Center for Integrated Breeding Research, University of Göttingen, Göttingen, Germany

**Keywords:** barley, high resolution mapping, powdery mildew, resistance locus, RLK

## Abstract

Powdery mildew caused by *Blumeria graminis* f. sp. *hordei* is a foliar disease with highly negative impact on yield and grain quality in barley. Thus, breeding for powdery mildew resistance is an important goal and requires constantly the discovery of new sources of natural resistance. Here, we report the high resolution genetic and physical mapping of a dominant race-specific powdery mildew resistance locus, originating from an Ethiopian spring barley accession ‘HOR2573,’ conferring resistance to several modern mildew isolates. High-resolution genetic mapping narrowed down the interval containing the resistance locus to a physical span of 850 kb. Four candidate genes with homology to known disease resistance gene families were identified. The mapped resistance locus coincides with a previously reported resistance locus from *Hordeum laevigatum*, suggesting allelism at the same locus in two different barley lines. Therefore, we named the newly mapped resistance locus from HOR2573 as *MlLa-H.* The reported co-segregating and flanking markers may provide new tools for marker-assisted selection of this resistance locus in barley breeding.

## Introduction

Cultivated barley (*Hordeum vulgare* ssp. *vulgare* L.), is ranked fourth after rice (*Oryza sativa* L.), wheat (*Triticum aestivum* L.) and maize (*Zea mays* L.) in terms of crop production. The prevalent use of barley is as a source of feed and forage for livestock, and as source for food and beverages for humans (Ullrich, 2010; Newton et al., 2011). According to FAO reports on global trade of barley and barley products, more than 20 million tons of barley grains are exported and imported annually worldwide, accounting for about US$3 billion. Nevertheless, losses due to pests and diseases in cereals continue to pose a substantial threat to agricultural food and feed production and impact economic decisions as well as practical developments. A cost effective and environmentally sustainable strategy to mitigate the damage and losses caused by plant pathogens is to deploy plant varieties possessing genetic resistance (Johnston et al., 2013). Unlocking genetic diversity in genebank collections is of prime importance to discover and deploy genetic resistance genes or alleles that have been lost during domestication and intensive selection in breeding programs (McCouch et al., 2013). The primary, freely crossable, gene pool of barley consists of cultivated barley including landraces and its direct wild relative *H. vulgare* ssp. *spontaneum* and provides a source of still un-used and valuable disease resistance alleles. In this regard, resistance phenotyping of barley genetic resources preserved in ex situ collections is a mean to identify the genetic basis of resistance and introduce it into modern barley cultivars.

Powdery mildew caused by *Blumeria graminis* f. sp. *hordei* (*Bgh*) is a foliar disease of barley with worldwide importance (Glawe, 2008). The relatively cool and humid climate of Europe fosters the spread of powdery mildew, making it the most prevalent European barley disease (Jørgensen and Wolfe, 1994) leading to yield losses of up to 30% and lower grain quality (Corrion and Day, 2001; Czembor, 2002). Until now, some of the previously identified powdery mildew resistance loci in barley have been exploited by plant breeders to develop resistant cultivars. In fact, all seven barley chromosomes harbor important powdery mildew resistance loci and still novel genes are continually being located to its chromosomes (Řepková et al., 2006). To increase the durability for barley powdery mildew resistance, breeders are continuously looking for new monogenic as well as polygenic resistance sources derived from diverse barley germplasm to improve resistance through gene pyramiding.

Previous QTL mapping studies conducted on barley powdery mildew resistance have suggested that the telomeric region of barley chromosome 2H represents an important genomic region for mildew resistance. Several significant QTLs near the distal end of this chromosome have been repeatedly been reported to be associated with powdery mildew resistance (von Korff et al., 2005; Marcel et al., 2007; Aghnoum et al., 2010; Schweizer and Stein, 2011). However, the confidence interval (cM) of the identified powdery mildew resistance QTLs in this region, regardless of rather large mapping population size (∼110–200 individuals) and high recombination frequency in this region (≤1.1 Mb/cM) according to Künzel et al. (2000); IBSC (2012), and Mascher et al. (2017), was too large to allow map-based cloning (St. Clair, 2010). In fact, the availability of sufficient number of SNPs was a limiting factor in marker development. The recent progress in next-generation sequencing (NGS) technologies provides the possibility of cost-effective high-throughput *de novo* SNP discovery within the genome and parallel genotyping (Deschamps and Campbell, 2010). Indeed, multiple individuals can be rapidly sequenced with low cost and the detected SNPs can easily be converted into individual molecular markers for further application or directly used in high-density linkage map construction (Ruperao and Edwards, 2015). However, for crops with medium to large genomes, where much of the sequence is repetitive and the proportion of gene space is limited, a reduced-representation strategy like genotyping-by-sequencing (GBS) is a cost effective approach to discover thousands of SNPs that can be directly used for high density linkage map construction, precise localization of the QTLs and further marker development (Elshire et al., 2011; He et al., 2014).

The diploid nature of barley (2n = 14) with high degree of inbreeding along with the ease of making genetic crosses made barley a favorable biological model for genetic and genomic studies (Saisho and Takeda, 2011). Consequently, comprehensive barley genomic resources have been developed to facilitate the analysis of the barley genome during the last two decades. The recently published barley reference-quality genome sequence (Beier et al., 2017; Mascher et al., 2017) and a variety of newly developed web-based tools providing barley genomic data (Colmsee et al., 2015) have facilitated many downstream applications in gene identification and isolation like positional gene cloning and comparative genomic analysis with other *Triticeae* (Mascher et al., 2017).

The main objective of the present study was to perform high resolution genetic and physical mapping of a major resistance locus segregating in the recombinant inbred line (RIL) population ‘HOR2573 × Morex.’ This was achieved through applying next generation sequencing-based strategies and taking advantage of the improved barley genomic resources infrastructure.

## Materials and Methods

### Plant Material and Phenotyping

An Ethiopian landrace ‘HOR2573,’ resistant to seven highly virulent powdery mildew isolates [three European (78P, D12-12, and CH4.8) and four Israeli (35, 69, 148, and 289) isolates, Supplementary File 1], was previously crossed to a six-rowed malting cultivar ‘Morex’ (susceptible to all the tested *Bgh* isolates). The phenotyping of the parental lines was performed through both detached leaf assay (seedling stage) and whole plant assay at field, revealing a strong correlation between resistance data from both assays (Spies et al., 2012).‘HOR2573’ responds to all the tested *Bgh* isolates by hypersensitive cell death in leaves detached from 2-week old plants. The Swiss field isolate, CH4.8, among the European isolates showed a decreased infection (less than 5% leaf area covered by colonies) – the same as the four Israeli isolates – on HOR2573, thus was selected for the further study. Ninety-five F_6_-RILs, derived by single-seed-descent (SSD) through five cycles of selfing from the cross of ‘HOR2573 × Morex,’ were used for phenotyping and genetic mapping in three independent experiments. Within each experiment, eight seeds per RIL (*F*_6:7_) were sown as eight biological replicates. Plants were phenotyped 14 days after sowing using the second seedling leaf in a detached leaf assay. For this purpose, the plants were grown in trays at 17–20°C under long day conditions (16 h) in the greenhouse. The middle part of the second leaf was cut into two 3 cm long pieces (technical replicates). Detached leaves were placed surfaces upward in four-column plates on water agar (1%) containing benzimidazole (40 mg/l) as senescence inhibitor. In each column of one plate, five RILs were located in randomized block design in combination with both positive (susceptible parent) and negative (resistant parent) controls. The prepared leaf segments were inoculated under the inoculation tower through blowing *Bgh* conidia (isolate CH4.8) of the sporulating leaves (from four sides) according to Altpeter et al. (2005), receiving final spore densities 20–30 conidia per mm^2^. The inoculated detached leaves were kept in the incubator growth chamber at 20°C, 60% humidity, 16 h light period and scored macroscopically at 7 days post inoculation (dpi). The disease intensity was rated based on infection area (%) according to Mains and Diktz (1930) and Kølster et al. (1986). Based on the infection area, the rating scores were finally grouped into two groups of resistant (classes 1 and 2, with less than 25% leaf infection area) and susceptible (classes 3 and 4, leaf infection area ≥25%) plants.

### Preparation of Genomic DNA

Plant material for DNA extraction was grown under standard greenhouse conditions (16 h day/8 h night, 20°C). Young third leaves were sampled and immediately transferred into liquid nitrogen. Genomic DNA was extracted using guanidine thiocyanate-based DNA isolation in 96-well plate format according to Milner et al. (2018). The DNA concentration of the samples was measured using Qubit^®^ 2.0 Fluorometer (Invitrogen, Carlsbad, CA, United States) according to the manufacturer’s protocol. For accurate DNA quantification of higher number of samples, Quant-iT^TM^ PicoGreen^®^ dsDNA assay kit (Invitrogen, Carlsbad, CA, United States) and a Synergy HT microplate reader (BioTek, Bad Friedrichshall, Germany) were used.

### Marker Development and Primer Design

The SNPs between resistant and susceptible genotypes identified through GBS assay, were converted into Cleaved Amplified Polymorphic Site (CAPS) markers using SNP2CAPS software (Thiel et al., 2004). Primers used for marker development were designed using the online software Primer3 v. 0.4.0^1^ (Koressaar and Remm, 2007; O’Halloran, 2015). Default parameters were used with minor modifications. Guanine-cytosine content (GC-content) was set within the range of 50–55% and the product size was adjusted according to the experimental requirement between 300 and 1,000 bp. The primer length was set between 19 and 21 bp and primer melting temperature (*T*_m_) was adjusted around 60°C. The restriction digestion reaction was performed according to manufacturer recommendations using a thermocycler for incubation. DNA fragments were separated on a 1.5% agarose gel for genotyping.

### PCR Amplification and Sanger Sequencing

The DNA amplification was performed on GeneAmp PCR Systems 9700 (Applied Biosystems, Darmstadt, Germany) using a standardized touchdown PCR profile with HotStarTaq DNA Polymerase (Qiagen, Hilden, Germany): initial denaturation for 15 min at 95°C, followed by four cycles of denaturation at 95°C/30 s, annealing at 62°C/30 s (decreasing by 1°C per cycle), extension at 72°C/60 s); then 35 cycles denaturation at 95°C/30 s, annealing at 58°C/30 s, extension at 72°C/60 s; followed by a final extension step at 72°C/7 min. Based on amplicon length, the extension time was modified (1 min/1 kb). The PCR products were resolved by 1.5–2.5% gel-electrophoresis depending on amplicon size. PCR products were purified using the NucleoFast 96 PCR Kit (Macherey-Nagel, Germany) and sequenced using BigDye Terminator chemistry (BigDye^®^ Terminator v3.1, Applied Biosystems, Darmstadt, Germany) on the 3730xl DNA Analyzer (Applied Biosystems, Carlsbad, CA, United States). Sequence analysis was performed using ‘Sequencher 4’ software (Genecodes Corporation, United States). The identified SNPs between resistant and susceptible genotypes were converted into CAPS markers according to procedures previously described in section marker development and primer design.

### GBS Library Preparation and Data Analysis

All 95 F_6_ RILs and the two parental genotypes ‘HOR2573’ and ‘Morex’ were pooled per lane in an equimolar manner and sequenced on the Illumina HiSeq 2500, 1×107 cycles, single read, using a custom sequencing primer following previously established procedures (Mascher et al., 2013; Wendler et al., 2014). Prior to library preparation, genomic DNA was quantified using PicoGreen (Invitrogen, Carlsbad, CA, United States) and normalized to 20 μl of 10 ng/μl (200 ng total) in 96-well plates. For quality control of DNA, the GBS library was analyzed with an Agilent 2100 Bioanalyzer (Agilent Technologies, Santa Clara, CA, United States) using the Agilent High Sensitivity DNA kit. Finally, the quantification control of the library was performed using qPCR according to Mascher et al. (2013).

The genotype calls from the sequencing data were filtered in order to select only SNPs matching the default criteria. The default parameters were defined for a RIL population by Mascher et al. (2013), considering the expected residual heterozygosity of 1–2% in the population presented in this study. In total, 46,689 and 15,798 SNPs were obtained genome-wide at minimum sequence read coverage of two- and six-fold, respectively. Furthermore, to reduce the computational errors in JoinMap^®^ 4.0, SNPs with more than 10% missing data were excluded from further analysis. This approach delivered 10,644 genome-wide SNPs at minimum two-fold read coverage. Of these, 1,843 SNPs were located on chromosome 2H. To make variant calls with a higher degree of confidence, a set of 1,394 genome-wide SNPs with robust variant calls (six-fold read coverage) were utilized to construct a genetic linkage map.

From GBS data, three plants were identified harboring a heterozygous region covering the resistance locus meaning that progeny of each of these plants segregate for the locus interval representing as a heterogeneous inbred family (HIF) according to Tuinstra et al. (1997). Heterozygosity of these three selected HIFs (HIF145, HIF567, HIF836) for the respective region was re-evaluated based on number of alternative allele coverage out of the total read coverage, confirming that selected plants were heterozygous for the target interval. The progeny of these three HIFs was used in the following for high resolution mapping of the locus according to Tuinstra et al. (1997). Consequently, an additional segregating *F*_7:8_ population consisting of 940 plants (the progeny of the identified heterozygous recombinants) was screened using flanking and co-segregating markers of the targeted interval to identify additional recombinants and increase the genetic resolution in the vicinity of the targeted locus.

### Genetic Linkage Analysis

JoinMap^®^ 4.0 software (Van Ooijen, 2006) was used for genetic linkage analysis following the instructions manual. Only genotype calls with at least six-fold read coverage were included to construct the genetic linkage map. A linkage map was produced by regression mapping using the Kosambi function. Markers were assigned to seven linkage groups based on Logarithm of Odds (LOD: >5) groupings and the linkage groups were assigned to barley chromosomes on the basis of the locus coordinates determined during read mapping against the barley reference genome assembly (Mascher et al., 2017).

### Statistics of the Phenotypic Analysis

The three independent phenotyping experiments were treated as three environments. The phenotypic data analysis was performed using the software ASReml-R 3.0 (Butler et al., 2009). The mean infection area in each experiment (considered as environment) was used to calculate the best linear unbiased estimates (BLUEs) with the following model:

$$y_{\mathrm{ijmno}}=\mu +g_{i}+l_{o}+{\left(\mathrm{gl}\right)}_{\mathrm{io}}+s_{\mathrm{jo}}+P_{\mathrm{jmo}}+c_{\mathrm{jmno}}+e_{\mathrm{ijmno}},$$

Where y_ijmno_ is the phenotypic performance of *i^th^* genotype in *n^th^* column of *m^th^* plate in *j^th^* inoculation tower of *o^th^* environment, μ is the intercept, *g_i_* is the effect of *i^th^* genotype, *l_o_* is the effect of *o^th^* environment, (*gl*)*_io_* is the interaction between *i^th^* genotype and *o^th^* environment, *s_jo_* is the effect of *j^th^* inoculation tower in *o^th^* environment, *p_jm_* is the effect of *m^th^* plate in *j^th^* inoculation tower of *o^th^* environment, *c_jmn_* is the effect of *n^th^* column in *m^th^* plate of *j^th^* inoculation tower in *o^th^* environment, and *e_ijmno_* is the error of *y_ijmno_*. For BLUEs estimation, only μ and *g_i_* were treated as fixed effects and for heritability estimation, all the effects were treated as random except μ.

The broad-sense heritability can be calculated with the following equation:

$$h^{2}=\frac{{\text{σ}}_{g}^{2}}{{\text{σ}}_{g}^{2}+\frac{{\text{σ}}_{\mathrm{gl}}^{2}}{\mathrm{Nr.env}}+\frac{{\text{σ}}_{e}^{2}}{\mathrm{Nr.env*Nr.rep}}}$$

### QTL Analysis

The QTL analysis was performed using GenStat v16 software (VSN International, Hemel Hempstead, Hertfordshire, United Kingdom). An initial genome-wide scan was carried out by simple interval mapping (SIM) to obtain candidate QTL positions. These were used as cofactors in subsequent scans (composite interval mapping, CIM). One or more rounds of CIM were performed, implying a genome-wide scan for QTL effects in the presence of cofactors, which were usually potential QTL positions detected at previous steps. Following back-selection from a set of candidate QTL, a final set of estimated QTL effects was obtained. The LOD significance threshold (α = 0.05) was estimated by 1,000 permutation tests. The 95% confidence interval was taken to be the chromosomal region where the LOD score has dropped less than one from the linkage peak (Sinha et al., 2009).

### Re-annotation of the Resistance Locus Interval

The automated annotation of the barley reference sequence (Mascher et al., 2017) might contain inaccuracies and could have missed genes, thus, the annotation of the *MlLa-H* interval was reassessed. For this purpose, the unique sequences of the target region were extracted using the Kmasker-web tool^2^. The re-annotation of the non-redundant sequences relied on nucleotide similarity search using BLASTN against the non-redundant DNA/protein database of NCBI. Moreover, the disease resistance genes identification approach published by Jupe et al. (2013) was performed using the established descriptive amino acid motifs in the motif alignment and search tool (MAST) to predict sequences with a motif composition similar to disease resistance analogs (RGAs).

In addition, some of the published annotated genes might miss sequences residing in start and stop codon regions. Therefore, to identify the putative start and stop codons for these gene models located in the interval where the *MlLa-H* gene resides, the protein sequence of each gene model was used to perform protein similarity search using BLASTP against the non-redundant protein sequence (nr) database. The protein sequence of the best hit from one of the closet species (rice, bread wheat, and *Aegilops tauschii*) was selected for alignment using TBLASTN against the barley reference genome to get the corresponding physical coordinates. Based on physical coordinates, as well as predicted open reading frames, the putative start and stop codons as well as exon and intron regions were determined. This allowed us to obtain the complete coding sequences of all the published annotated genes in the delimited target interval (Mascher et al., 2017).

## Results

### Phenotypic Data Analysis

In all three phenotyping experiments, the resistant parent ‘HOR2573’ always had the highest resistance score (≤2.5% leaf infection area, class 0) whereas maximum susceptibility was always recorded for ‘Morex,’ the susceptible parent (≥80% leaf infection area, class 3) documenting high inoculation/infection efficiency. The broad sense – Heritability for powdery mildew resistance was higher than 98% in all three independent phenotyping experiments, indicating that most of the phenotypic variation was genetically determined. Significant correlations were observed among all three phenotyping experiments with *r* between 0.91 and 0.94 in each couple of experiments, indicating high infection efficiency in all the three experiments.

### Genotyping of the RIL Population and Construction of Genetic Linkage Map

The reduced set of 1,394 SNPs (six-fold read coverage) was used to construct a genetic linkage map with seven linkage groups (LOD = 5.0) comprising between 154 (1H) and 269 (5H) markers, which were distributed evenly on each chromosome. The marker density varied from 1.1 for chromosome 4H (137 SNPs/119.7 cM) to 1.9 for chromosome 2H (252 SNPs/134.4 cM). The accuracy of the genetic linkage map was checked through the observed consistency between genetic marker positions and their respective physical order in the reference genome sequence (Mascher et al., 2017). The genetic map length ranged between 119.7 cM (4H) and 171.8 cM (7H) per chromosome, respectively, with a total map length of 1,000 cM, which is in the similar range as reported for other genetic maps of barley (Stein et al., 2007; Close et al., 2009; Mascher et al., 2013).

### QTL Mapping for Powdery Mildew Resistance

**FIGURE 1:**
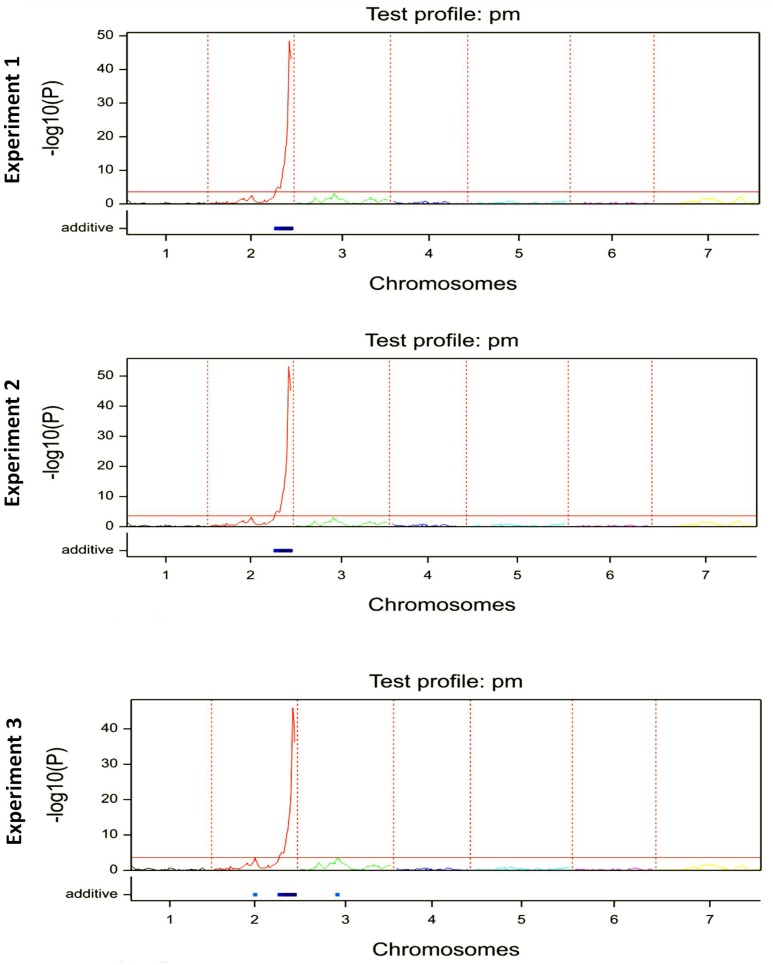
The QTL mapping analysis for Bgh resistance in F6:7 generation of ‘HOR2573 x Morex’ population in each phenotyping experiment/environment.

Linkage analysis for single trait in single/multiple environment(s) for both Interval Mapping and CIM methods revealed the same QTL with LOD peaks of 48, 53, and 46 on the long arm of chromosome 2H for all three environments, respectively (Figure 1).

The QTL interval was stable across all environments explaining an average of 73.3% of the phenotypic variance in the first, 74.7% of the phenotypic variance in the second and 71.4% of the phenotypic variance in the third environment (Table 1). The detected single major effect QTL was assigned to an interval of 3 cM with 95% confidence flanked by markers M238 and M252.

**Table 1:** Summary of QTL found for *Bgh* resistance in *F*_6:7_ generation of ‘HOR2573 × Morex’ population.

Exp./Env.	Chromosome	Markers_interval^1^	Interval size (bp)^2^	LOD score	*R*^2∗^	Additive effect^3^ (infection area %)
1	2H	M238_M252	3,482,164	48.55	0.73	-17.36
2	2H	M238_M252	3,482,164	53.16	0.75	-17.62
3	2H	M238_M252	3,482,164	45.97	0.71	-17.23

*^1^ 95% confidence interval is supported by LOD = 25. ^*2*^The physical coordinates of the 95% confidence interval flanked by markers M238 and M252 on barley reference genome: 762,829,007 and 766,311,171 bp, respectively. ^*3*^The negative value of additive effect means that a single allele of resistant parent ‘HOR2573’ decreases the infection area according to the value. ^∗^The remaining 27% of phenotypic variation which is not explained by this QTL might be due to random error, all other non-genetic variation (like unavoidable inaccuracies in estimating % leaf infection severity, the position of leaves on the plates, a bit heterogeneous inoculum deposition) and other genetic variation, like statistically non-significant QTLs that did not pass the threshold line for declaring statistical significance of a QTL which is obtained through permutation tests*.

The strength and the effect of the identified QTL on phenotypic variation suggested that the powdery mildew resistance from ‘HOR2573’ was most likely controlled by a single major gene. To validate this possibility, disease scoring was re-performed with two qualitative classes (resistant vs. susceptible class) independently from the previous phenotyping scores in order to obtain unbiased results. Based on the qualitative evaluation, 51 out of 95 RILs were consistently scored as resistant whereas 44 RILs were scored as susceptible plants indicating the inheritance of a monogenic Mendelian factor [1:1, *X*^2^ = 0.5156 < 3.841 at the certainty level of (1 -*P*-value = 0.95) with the degrees of freedom (d.f. = 1)]. These results corroborated the presence of a single major effect dominant locus/gene controlling powdery mildew resistance in the population ‘HOR2573’ × ‘Morex.’

**FIGURE 2:**
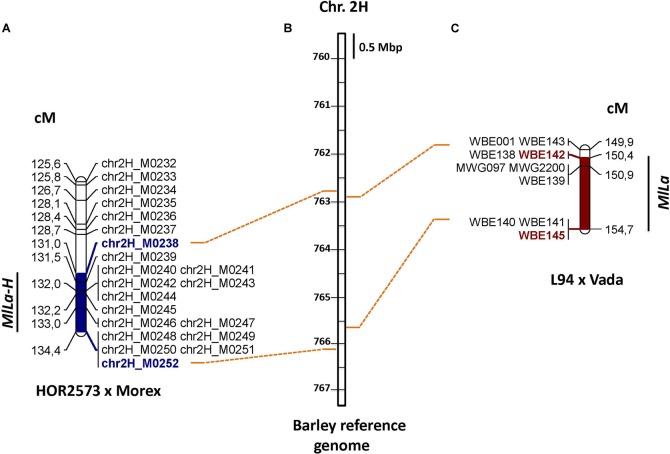
**(A)** The location of powdery mildew resistance MlLa-H locus on chromosome 2 in the population ‘HOR2573 × Morex’ **(B)** The position of the powdery mildew resistance MlLa locus on chromosome 2 in the population L94 × Vada **(C)** Anchoring of flanking markers of these two loci on barley reference genome.

The long arm of chromosome 2H is known to carry the *‘Laevigatum’* resistance locus (known as *MlLa*) (Marcel et al., 2007). To assess the possibility of an overlap between the QTL detected for ‘HOR2573’ × ‘Morex’ and the *MlLa* locus, flanking and co-segregation genetic markers were used for BLAST searches against the barley reference genome. All *MlLa* locus associated markers (WBE142, WBE138, MWG2200, WBE141, and WBE145) could be anchored to the M238-M252 interval (Figure 2), suggesting that either two independent physically closely linked genes or alleles of the same locus might explain powdery mildew resistance in ‘Vada’ (derived from ‘*Laevigatum’*) and in ‘HOR2573.’ To point out this coincidence clearly, we propose to name the ‘HOR2573’ mildew resistance locus *MlLa-H*, indicating that the resistance-conferring putative *MlLa* allele of this study was derived from the Ethiopian landrace ‘HOR2573.’

### High Resolution Genetic Mapping of the 2HL Resistance Locus

For high resolution genetic mapping of the resistance locus 1,001 progeny plants of three F6 HIFs were used. The resistance evaluation of HIF-population with the same *Bgh* isolate, CH4.8, resulted in the identification of 742 resistant and 259 susceptible plants, consistent with the segregation of a single dominant gene (3:1, *X*^2^ = 0.407 < 2.706 and *P*-value = 0.1; degrees of freedom d.f. = 1). This segregation pattern was also evaluated individually in each HIF family (HIF145, HIF567, HIF 836), verifying the monogenic dominant inheritance of the *MlLa-H* locus (Table 2).

**Table 2:** Phenotypic segregation of powdery mildew resistance in each of heterozygous inbred families.

Family	Number of resistant plants	Number of susceptible plants	*X*^2^ *_0.1_*
HIF145	369	117	0.22
HIF567	205	74	0.34
HIF836	168	68	1.83

*H_0_= 3 (number of resistant plants): 1 (number of susceptible plants). H1 = H0 is false, *P*-value = 0.1 and d.f. = 1; the calculated χ^*2*^ value is too low to reject the hypothesis*.

**FIGURE 3:**
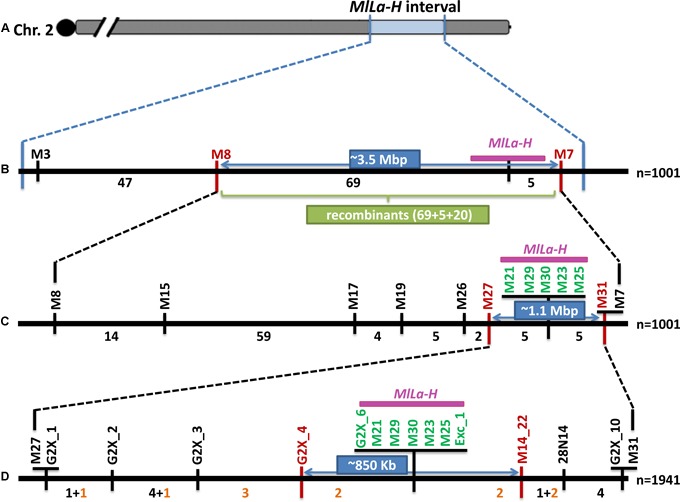
High resolution mapping of the powdery mildew resistance locus *MlLa-H.* **(A)** The genomic region containing the *MlLa-H* locus identified through QTL analysis is shown in blue. **(B)** The identification of 141 recombinants and mapping of the *MlLa-H* locus between M8 and M7. The green box presents the number of recombinants between M8 and M7, of which 74 occurred between marker and phenotype, 20 recombinants occurred from heterozygous to homozygous resistance. **(C)** The reducing of target interval to 1.1 Mbp and remaining 10 recombinants at this interval. **(D)** Identification of additional 11 recombinants through screening of additional 940 F_2_-like individuals by the flanking markers M27, M31 plus co-segregating markers (M21 and M25). **(D)** Narrowing down of the target interval to 850 kb. In each step the flanking markers are highlighted in red. The physical distance between two flanking markers is written in dark blue box. The co-segregating markers with the phenotype (the target locus is shown in pink) are highlighted in green. The number of recombination events between markers is shown below the black line which presents the barley reference genome. The identified additional 11 recombinants are highlighted in orange.

For genotyping of 1,001 plants, three CAPS markers (M3, M8, and M7 in order of appearance in this interval, see Figure 3A) were developed by taking advantage of GBS-derived SNPs within the locus interval. A total of 141 recombinants were identified between the three selected markers (Figure 3B) placing the resistance locus between M8 and M7, in close proximity to M7. Further marker saturation reduced the interval to 1.1 Mbp (10 recombination events), flanked by markers M27 and M31 (Figure 3C). An additional 940 progeny plants of the identified heterozygous recombinants were screened by utilizing the closest flanking markers (M27 and M31) plus two markers previously co-segregating with the resistance locus. This identified additional 11 recombinants for this interval. Eventually, the target interval was reduced to 850 kb, flanked by marker G2x_4 and M14_22, containing only two recombinants at either side of the resistance locus which was co-segregating with a cluster of seven markers (Figure 3D). The sequence information of all key markers can be found in Supplementary File 2.

### Identification of Candidate Genes for the MlLa-H Interval

The attempts of re-annotation of the *MlLa-H* interval in the ‘Morex’ reference sequence confirmed the automated annotation of the barley reference sequence (Mascher et al., 2017) and the presence of no additional genes/ORFs in the corresponding region. Based on automated annotation of the *MlLa-H* interval, seven high confidence (HC) genes (*HORVU2Hr1G126250, HORVU2Hr1G126290, HORVU2 Hr1G126350, HORVU2Hr1G126380, HORVU2Hr1G126440, HORVU2Hr1G126510*, and *HORVU2Hr1G126540*) are located within the genetically delimited target interval. In addition, the structural annotation of the seven genes predicted for the *MlLa-H* interval was validated through sequence comparisons to putatively orthologous genes from closely related species. This showed that the automated gene annotation for four gene models (*HORVU2Hr1G126290, HORVU2Hr1G126380, HORVU2Hr1G126510*, and *HORVU2Hr1G126540*) had incomplete coding sequences in the automated annotation and thus those were revised accordingly.

**FIGURE 4:**
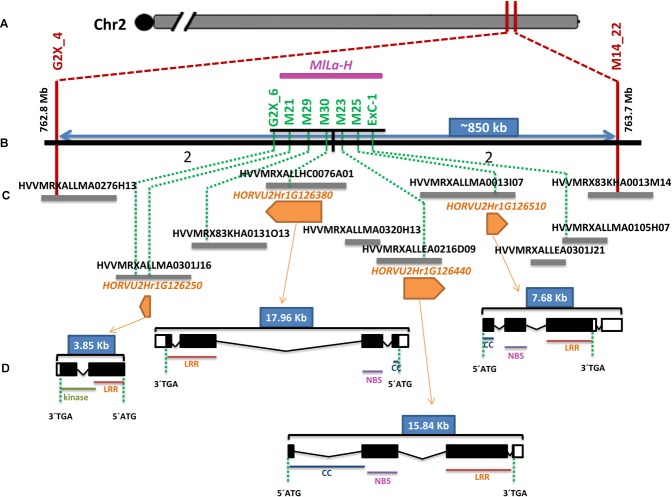
Physical organization and gene content of the ‘Morex’ *MlLa-H* locus. **(A)** The genetically and physically delimited *MlLa-H* locus is indicated by two red vertical lines standing for flanking markers. **(B)** Expanded view of the delimited target interval on the barley reference genome (black solid line). The number of recombination events between flanking marker at either side of co-segregating markers (highlighted in green) is written below the black line. **(C)** The overlapping BAC clones spanning the *MlLa-H* interval based on minimum tilling path (MTP) are shown as gray bars. The position of each DNA marker on BAC clones is shown by dashed green lines. The four resistance genes in the interval are shown in orange pentagons with the corresponding ID above them. The direction of pentagons shows which strand of DNA was sequenced representing the direction of each gene on the reference genome. **(D)** The structure of each R gene model in cv. ‘Morex’ (the barley genome reference and as the susceptible parent in this study) is represented in black (exon) and white (untranslated regions) boxes. The distance between boxes represents the introns. The size of each gene is written in blue boxes above of each gene model. The corresponding protein domains are written below exons.

Powdery mildew resistance conferred by the *MlLa-H* locus, derived from ‘HOR2573,’ is dominantly inherited, involves a hypersensitive response-like programmed cell death at a microscopic level, thus it is most likely race-specific. This pattern of resistance may suggest the involvement of a gene belonging either to the Nucleotide Binding Site Leucine Rich Repeat (NBS-LLR) or Receptor Like Kinase (RLK) gene families, therefore it was anticipated that the delimited target interval (∼850 kb) of the *MlLa-H* locus would contain candidate genes belonging to the expected classes of resistance genes. From the seven HC genes, four gene (*HORVU2Hr1G126250, HORVU2Hr1G126380, HORVU2Hr1G126440*, and *HORVU2Hr1G126510*) in the delimited target interval were RGAs, one gene (*HORVU2Hr1G126250*) belongs to RLK gene family and the rest to the NBS-LLR family (Figure 4). In context of dominant race-specific resistance this qualified each of them as candidate genes for the *MlLa-H* locus.

Based on the assigned functional annotation of genes predicted in the barley reference sequence and publicly available transcriptome and gene expression profiling data from different plant tissues in barley (IBSC, 2012), none of the three remaining gene models (*HORVU2Hr1G126290, HORVU2Hr1G126350, HORVU2Hr1G126540*) is suggested of having a role in resistance to plant pathogens or plant/pathogen interaction; therefore, they were excluded for any further analysis. As detailed information, *HORVU2Hr1G126290* is an uncharacterized protein. Its protein sequence similarity search against the orthologous genes in other species indicated that it is also uncharacterized in other species. In addition, a survey on publicly available transcriptome and gene expression profiling data in barley (IBSC, 2012) showed that the *HORVU2Hr1G126290* is only expressed in tissues taken from developing grains, palea and rachis, suggesting that it does not play a role in regulating disease resistance. Likewise, the survey on publicly available gene expression data for *HORVU2Hr1G126540* (homology with Amidase superfamily) showed that it is also highly expressed in developing grains, presenting no role in plant immune responses. The third gene model, *HORVU2Hr1G126350* has high homology with SCAR family, being involved in plant cell morphogenesis such as controlling cell division and elongation. Therefore, based on the functional annotation or the transcript evidence, these three genes can be ruled out as candidates.

### Re-sequencing of Candidate Genes in ‘HOR2573’ and ‘Morex’

The four RGAs detected at the *MlLa-H* locus were all promising candidates for the resistance gene. Sequence comparison in resistant (*MlLa-H*) and susceptible (*mlLa-H*) genotypes should provide help prioritizing among the four candidate genes. In fact, the presence of a polymorphism correlated to the phenotype in a gene re-sequenced from resistant parent, can make it a good candidate, whereas either the absence of non-synonymous polymorphisms or the emergence of premature stop codons in each RGA in ‘HOR2573’ compared to ‘Morex’ is a clue to reject it as a candidate gene.

**FIGURE 5:**
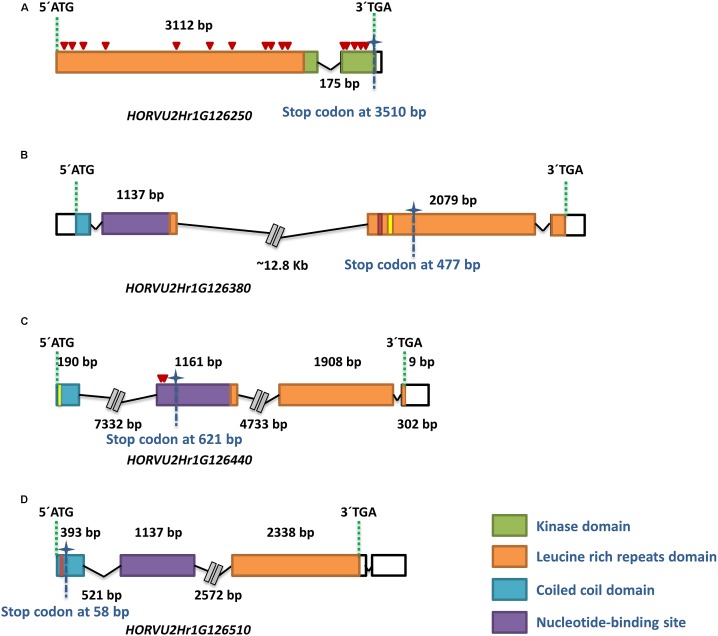
Characterization of the four potential candidate genes in the *MlLa-H* interval through re-sequencing in the resistant parent ‘HOR2573.’ The structure of each gene model in ‘HOR2573’ is shown **(A–D)**. All four resistance genes were sequenced from the resistant parent genome. The PCR primers were designed from the corresponding gene in cv. ‘Morex.’ The ID of each gene model is written under gene structure. The colored and white boxes represent exons and UTRs, respectively. The color code green, orange, violet and blue display the protein domains standing for kinase, leucine rich repeats, nucleotide-binding site and coiled coil domains, respectively. The size of each exon is written above the boxes. The distance between boxes shows the intron size. For the large intron sizes, the distance has been truncated. Premature stop codons are indicated by asterisks with identified position at the bottom of vertical dashed line. The non-synonymous SNPs are indicated by red triangles. Insertions and deletions are depicted with vertical red and yellow lines.

All four genes could be amplified in their entire length from both ‘Morex’ and ‘HOR2573.’ The re-sequencing from susceptible parent ‘Morex’ always confirmed the sequence information of the published reference sequence. The sequence comparison of the first RGA (*HORVU2Hr1G126250*) between parental lines revealed 20 SNPs, including 4 synonymous and 16 non-synonymous SNPs, leading to amino acid changes in both LRR and kinase domains (Figure 5A). The re-sequencing result of the second RGA (*HORVU2Hr1G126380*) from resistant parent’s genome ‘HOR2573’ showed that a 42 bp deletion plus a 53 bp insertion occurred in different parts of the second exon, corresponding to the LRR domain. These deletion and insertion led to a premature stop codon (Figure 5B) and a probable loss of function of the domain, suggesting it represents a pseudogene in the resistant genotype. The comparison for the third RGA, *HORVU2Hr1G126440*, revealed the presence of two paralogs of this gene in ‘HOR2573,’ the resistant genotype. One copy is 100% identical to the ‘Morex’ allele (not shown in the Figure 5) and likely represented the orthologous gene. The other copy (putative paralog) showed several SNPs in the exons plus a single bp insertion in the first exon and two consecutive nucleotide changes in the second exon predicted to induce a frame-shift, thus, the *HORVU2Hr1G126440* paralog of ‘HOR2573’ likely represents a pseudogene. Furthermore, the comparison of the predicted protein sequences from resistant and susceptible parents suggested that due to the frame shift, the LRR domain was absent, leading to loss of function status of this gene model in ‘HOR2573’ (Figure 5C). Two paralogs were also observed in ‘HOR2573’ for the RGA *HORVU2Hr1G126510.* One copy was 100% identical to the ‘Morex’ allele (not shown in the Figure 5) and likely represented the orthologous gene whereas the putative ‘HOR2573’ paralog carried a 4 bp deletion in the first exon, leading to a frame shift and pre-mature stop codon, thus, the *HORVU2Hr1G126510* paralog also likely represents a pseudogene (Figure 5D).

Although there is no further information on the location of the second copy gene, it is highly expected both NLR and RLK with the highest sequence identity (putative tandem duplications or local paralogs) to be typically found in close physical proximity and are the result of tandem duplication events (Baumgarten et al., 2003; Cantalapiedra et al., 2016).

From these results *HORVU2Hr1G126250* was favored as the primary candidate gene for *MlLa-H* based resistance since it exhibited 4 synonymous and 16 non-synonymous SNPs, leading to amino acid changes in both the LRR and kinase domains in the resistant vs. the susceptible genotypes and was the only of the four candidate genes in ‘HOR2573’ and/or their respective paralogs that was not modified into a pseudogene.

## Discussion

### Fine Mapping Allowed to Map the *MlLa-H* Locus in a 850 kb Interval

In this study, we took advantage of improved barley genomic resources and state-of-the-art sequencing-based technologies to gain insights into a resistance locus called *MlLa-H*, located distally on barley chromosome 2HL. The locus corresponds to the interval of *MlLa*, derived from *H. laevigatum*, a previously reported powdery mildew resistance gene based on hypersensitivity response (Hilbers et al., 1992; Giese et al., 1993 Backes et al., 2003; Marcel et al., 2007). Due to its intermediate reaction type – the *MlLa* locus has garnered much attention from barley breeders and was immediately introduced into the modern barley varieties ‘Minerva’ and ‘Vada’ (Dros, 1957). The current study highlights the use of GBS technology for the construction of a high-density linkage map of the *MlLa-H* locus placing the locus within an 850-kb region carrying four disease resistance gene analogs. One gene belongs to the RLK and the rest to the NBS-LLR gene family making each of them a potential candidate gene for the *MlLa-H* locus. The observed physical to genetic distance ratio at the *MlLa-H* locus (∼1.16 Mb/cM) and physical distance of flanking markers would require screening of an additional 6,000 meioses (based on the formula of Dinka et al., 2007) to provide a chance of observing any additional recombination events between the cluster of the remaining co-segregating markers and the resistance gene to provide genetic evidence for rejecting several of the found candidate genes. The possibility of resolving the correct candidate gene by genetics and recombination, however, remains a theoretical option, since the candidate genes were observed in a susceptible genotype.

### A Gene Encoding LRR-RLK Protein as the Best Candidate Gene in the *MlLa-H* Interval

The conducted survey on publicly available gene expression data (IBSC, 2012) of the *HORVU2Hr1G126290, HORVU2Hr1G126350*, and *HORVU2Hr1G126540* clearly showed that these genes were expressed in palea, rachis and developing grains, implying that these genes might not play a role in plant immune responses, in this case in hypertensive response (HR). The re-sequencing analysis of three out of four potential candidate R genes within the *MlLa-H* interval from ‘HOR2573,’ the resistant genotype, displayed functional polymorphisms from SNPs to medium and/or large-scale insertions and deletions leading to premature stop codons compared to susceptible parent cv. ‘Morex’ (Table 3). These findings exclude those three genes as candidate genes for the *MlLa-H* locus, as all these polymorphisms are likely to lead to loss of function of the genes in the resistant genotype. In *HORVU2Hr1G126380*, predicted to encode an NBS-LRR gene, out-of-frame deletions or insertions were observed in the LRR domain leading to the premature stop codon and a probable loss of function of the domain. LRR domains in R genes have a specific function as site of protein–protein interaction for the recognition of pathogen effectors (Dangl and McDowell, 2006; Ye et al., 2017). Previous studies showed that the LRR domain and its sequence are essential for the recognition of the pathogen, and a mutation in different motifs of LRR domain in R genes could change the gene function either to the partial or complete loss of function of NB-LRR genes (Warren et al., 1998). Gassmann et al. (1999) showed that the transformation of the genomic sequence of Arabidopsis *RPS4*, a member of NBS-LRR family conferring resistance to *Pseudomonas syringae* pv. Tomato strain, causing premature stop codons in the LRR domain impeded the function of *RPS4*. It is then unlikely that a NBS-LRR gene with a severely truncated LRR domain would be a resistance gene. A similar situation has been also observed in *HORVU2Hr1G126510*, in which the presence of an early premature stop codon occurred in the CC domain, the first functional domain of the protein, making it a pseudogene. In *HORVU2Hr1G126440*, a premature stop codon occurred in the NB domain anticipated to cause loss of function as this is usually a highly conserved domain and is involved in signal transduction cascades (Tan and Wu, 2012).

**Table 3:** Summary of sequence analysis of four disease resistance analogs within target interval from ‘HOR2573.’

Gene ID	SNP position	SNP Morex/HOR2573	aa change/frame shift	Note
*HORVU2Hr1G126250*	71	C/T	Pro/Ser	EXON 1
**LRR-RLK gene family**	163	A/C	Arg/Ser	EXON 1
	254	C/G	Arg/Gly	EXON 1
	517	T/C	–	EXON 1
	705	G/A	Ser/Asn	EXON 1
	877	G/A	–	EXON 1
	1399	T/C	–	EXON 1
	1667	A/G	Lys/Glu	EXON 1
	2343	C/T	Thr/Ile	EXON 1
	2519	G/T	Val/Leu	EXON 1
	2819	C/T	His/Tyr	EXON 1
	2840	T/C	Tyr/His	EXON 1
	2904	G/C	Gly/Ala	EXON 1
	2978	A/G	Asn/Asp	EXON 1
	3188	A/G	Arg/Gly	EXON 2
	3220	A/T	Glu/Asp	EXON 2
	3344	T/G	Leu/Glu	EXON 2
	3345	T/A	–	EXON 2
	3372	T/C	Met/Thr	EXON 2
	3384	G/A	Arg/His	EXON 2
*HORVU2Hr1G126380* **NBS-LRR gene family**	364–406	42 bp deletion	Frame shift and pre-mature stop codon	EXON 2
	511–564	53 bp insertion		EXON 2
*HORVU2Hr1G126440* **NBS-LRR gene family**	29	1 bp insertion	Frame shift and pre-mature stop codon	EXON 1
	30	C/G		EXON 2
	31	G/C		EXON 2
*HORVU2Hr1G126510* **NBS-LRR gene family**	45–49	4 bp deletion	Frame shift and pre-mature stop codon	EXON 1

Interestingly, the sequencing results of these four R genes from the resistant parent pointed toward *HORVU2Hr1G126250* to be the most likely candidate for the *MlLa-H* locus. From structural annotation, this gene belongs to the Receptor-Like Serine/Threonine Kinase (RSTK) gene family; meaning that it contains an extracellular region, a single membrane spanning domain and an intracellular kinase domain (Becraft, 2002). The major group of RSTK contains an LRR domain, the extracellular region that is recognized by the repeated sequence LxxLxLxxNxLxx. A typical LRR belongs to the 3, 6, 12, or 24 repeat subfamily of LRR (Kajava, 1998). The structural annotation of *HORVU2Hr1G126250* suggested that this gene contains an extracellular LRR domain with six repeats. The resistant parent’s genome contains 4 synonymous and 16 non-synonymous SNPs for this gene compared to ‘Morex,’ leading to amino acid changes in both LRR and kinase domains. Among the four R genes in this cluster, this gene is the only one with meaningful non-synonymous polymorphisms. The study of divergence between ancestral copies of LRR-RLK suggested that some LRR-RLK characterized by fixation of a higher number of non-synonymous than synonymous mutations at some amino acid sites, highlighting the emergence of probably new advantageous functions for these R genes (Dufayard et al., 2017). It has been reported that both LRR and kinase domains are under different selective pressures according to their roles in resistance response. The LRR domain often undergoes a diversifying selection phase, obtaining new advantageous genetic variants, most likely in order to recognize the new virulent pathogen effectors, while the kinase domain is typically under purifying/negative selection leading to the removal of alleles that are deleterious such as functional and structural restrictions involved in signal transduction (Zhang et al., 2006).

Although the comparative sequencing analysis of the putative candidate genes in this target interval provided clear support for the hypothesis of *HORVU2Hr1G126250* representing the gene for the *MlLa-H*, further investigations are required to determine the gene function. This could either rely on (i) transgenic complementation through over-expression of the candidate resistance gene in a susceptible genotype (Ihlow et al., 2008), (ii) by stable or transient RNA interference (RNAi) or gene silencing (TIGS) (Douchkov et al., 2005), or by (iii) RNA guided CAS9 based site-directed mutagenesis or gene-editing (Lawrenson et al., 2015). All three approaches are established routines in barley, thus validation of gene function of *MlLa-H* candidate genes will be a feasible task.

Even if *HORVU2Hr1G126250* is providing a solid candidate gene, it cannot be ruled out that resistance is provided by presence/absence variation (PAV) of a resistance gene between resistant and susceptible genotypes, meaning that the candidate gene might be missing from the ‘Morex’ genome. Several studies have underlined the high probability of identification of PAV between genotypes with contrasting phenotypes.

Recently, Cantalapiedra et al. (2016) analyzed the exome sequences of three recombinant lines with contrasting resistance phenotypes from a high-resolution mapping population. By narrowing the position of the resistance derived from a Spanish landrace – showing a wide array of powdery mildew isolates – down to a single physical contig, they found large differences between the resistant lines and cultivar Morex as the reference genome, in the form of PAV in the composition of the NBS-LRR cluster. This finding suggested that the functional polymorphism in an R gene locus can occur from PAV of genes. The structural variation might also be constituted by a variable number of homologs in each haplotype which is the most prevalent PAV in multigene loci (Bergelson et al., 1998). The structural comparison of *Rpp5*, a multigene locus in a downy mildew resistant *Arabidopsis* ecotype, Landsberg erecta (Ler) with a susceptible ecotype, Columbia (Col-0) revealed the presence of ten *Rpp5* homologs in the entire Ler haplotype, whereas *Rpp5* haplotype in *Col-0* consisted of eight homologs. Bergelson et al. (1998) proposed the *Rpp5* locus contained dynamic gene clusters with capability to adapt fast to a new pathogen variant through modification of recognition regions, implying that these regions have been most likely undergone a diversifying and purifying selection (Noël et al., 1999). The structural analysis in both *Rpm1* and *Rpp5* clearly showed this variation was directly associated with the phenotype. To obtain the complete DNA sequence of the *MlLa-H* interval from the resistant parent, either developing high-quality *de novo* assembly from the flow-sorted barley mutant chromosome 2H or performing Targeted Locus Amplification (TLA) approach is highly recommended (de Vree et al., 2014; Thind et al., 2017; Bettgenhaeuser and Krattinger, 2018).

To sum up the current study, the reported high resolution mapping and physical delimitation of the resistance locus *MlLa-H* represents the fundamental steps for map-based cloning of the respective gene. The fine mapping of the target interval revealed the presence of four disease resistance gene homologs belonging to RLK and NBS-LLR gene families at this locus, which are the potential candidate genes for the race-specific resistance phenotype. The comparative sequencing analysis of these putative candidate genes between resistant and susceptible parents strongly suggested *HORVU2Hr1G126250* as being the best candidate gene. To validate this will require additional efforts like mutagenesis or transgene analysis for complementation or knock-out through gene-editing. Compared to quantitative resistance (conferred by several genes with small effects), the race-specific resistance is rather less challenging to incorporate into breeding programs, however, it is often not durable because of rapid changes in the pathogen virulence (Parlevliet, 2002). Combining multiple highly effective R genes, each covering a broad race spectrum, with many known successes is a practical approach to prevent or delay the development of boom-and-bust cycles commonly observed in the deployment of single R genes. The identified co-segregating and closest flanking markers though provide already new possibilities for marker-assisted selection of the *MlLa-H* locus in barley breeding.

## Author Contributions

PH performed the experimental work and performed data analysis, and wrote the manuscript. RZ contributed to data analysis. MM supported data analysis. AH conducted GBS sequencing. RN provided molecular marker information regarding the resistance locus derived from *Laevigatum’*, provided all relative information for the *Laevigatum’* locus. PS provided mapping population and overall supervision of phenotyping. NS designed the study, supervised the experimental work, and contributed to the writing of the manuscript. All authors read, corrected, and approved the manuscript.

## Conflict of Interest Statement

The authors declare that the research was conducted in the absence of any commercial or financial relationships that could be construed as a potential conflict of interest.

## Footnotes

1 http://bioinfo.ut.ee/primer3-0.4.0/

2 http://webblast.ipk-gatersleben.de/kmasker/
